# Viscoelastic Behavior of Crude Oil-Gum Emulsions in Enhanced Oil Recovery

**DOI:** 10.3390/polym14051004

**Published:** 2022-03-02

**Authors:** Mamdouh T. Ghannam, Mohamed Y. E. Selim, Abdulrazag Y. Zekri, Nabil Esmail

**Affiliations:** 1 Department of Chemical and Petroleum Engineering, Faculty of Engineering, United Arab Emirates University, Al-Ain P.O. Box 15551, United Arab Emirates; a.zekri@uaeu.ac.ae; 2 Department of Mechanical Engineering, Faculty of Engineering, United Arab Emirates University, Al-Ain P.O. Box 15551, United Arab Emirates; mohamed.selim@uaeu.ac.ae; 3 Department of Mechanical Engineering, Concordia University, 1455 de Maisonneuve Boulevard W., Montreal, QC M3G 1M8, Canada; esmail@encs.concordia.ca

**Keywords:** crude oil, xanthan, creep, recovery, polymer, emulsion

## Abstract

The experimental study of the Creep-recovery examination is necessary to understand the viscoelastic behavior of crude oil-Xanthan gum emulsions. The experimental measurements and analysis of these tests were completed using RheoStress RS100 under controlled stress CS-mode. Rheometers with CS-mode allow for a useful and direct technique for the experimental measurements of creep and recovery stages. This investigation covers a wide range of crude oil concentration of 0–75% by volume, Xanthan concentration range of 0–10^4^ ppm, and two types of Xanthan gums are used and investigated. The creep-recovery measurements of crude oil-Xanthan gums emulsions were extensively investigated. It was important to find the linear viscoelastic range for the examined crude oil-Xanthan gum emulsions. The experimental measurements and analysis of the creep-recovery examinations showed that the linear viscoelastic range was up to 1 Pa. The experimental investigation showed that the higher the concentration of the used gum and crude oil, the lower the compliance of the emulsions. For the Xanthan concentrations of less than 10^3^ ppm, the crude oil-gum emulsion exhibited viscous behavior. However, for the Xanthan concentration of higher than 10^3^, the examined emulsions displayed viscoelastic behavior.

## 1. Introduction

A considerable amount of crude oil—around half of the original magnitude—cannot be produced by traditional methods. Thus, enhanced oil recovery (EOR) is a very effective approach to extract the retained oil in an oil well [1]. There are many techniques that can be utilized to achieve this goal; some of these methods are alkali and surfactants as reported by Wylde et al. (2013) [2], and carbon dioxide injection [3,4]. Arjmand et al. (2012), Wang et al. (2013) and Wei et al. (2018) reported that the injection of polymer solution into oil wells is one of the effective techniques to capture a significant amount of the trapped oil in place [5,6,7]. Polymers can be used to modify the rheological properties of the pumped solutions to increase mobility ratio and therefore the crude production rate [8]. Polymer injection into crude oil wells is a useful technique of cultivating more crude oil through increasing the viscosity of the solution [9].

Polysaccharide biopolymer is one of the polymers that are widely employed in polymer flooding. Xanthan gum is a high molecular weight polysaccharide, which plays an important role in essential industries such as food and pharmaceutical [10]. Kamal et al. (2015) and Agi et al. (2018) revealed that many biopolymers may be injected in an oil well, whereas the Xanthan option is the highest utilized biopolymer. Xanthan gum is stable at high temperature and salinity, in-expensive, non-toxic, and biodegradable [11,12]. Therefore, Xanthan gum provides many attractive characteristics such as forming aqueous solution of high viscosity even for low gum concentration and chemically stable to function at well conditions [13]. Xanthan gum has been investigated by chemical and physical studies [14,15]. Rodd et al. (2000) reported that the aqueous solutions of Xanthan gum are widely studied for its flow behavior characteristics [16]. Xanthan solutions exhibit strong pseudoplastic behavior with significant suspending properties, which improve Xanthan characteristics for different applications such as nutrition, outlooks, medications and crude oil industries [16,17]. Whistler and BeMiller (1997) reported that the Xanthan gum aqueous solutions showed strong pseudoplasticity behavior which resulted from the development of high molecular weight collections of rigid molecules [18]. The Xanthan molecules in the well-arranged structure would reinforce the polysaccharide solution [19]. Sato et al. (1984) found that the polysaccharide molecules perform as a stiff shape at lower molecular weight, while at higher molecular weight the molecules modify to wormlike coils [20].

To reveal the viscoelastic properties of emulsions consists of crude oil and Xanthan material, creep-recovery investigation is one of the very useful techniques that can be employed. This test allows us to study the deformation response of a sample when a constant stress is applied versus elapsed period, as explained by Ferry (1980). Examined materials that exhibit viscoelastic behavior are comprised of stretched chain molecules, which mix and coil together to form a complex structure at a low energy level [21]. Through the distortion phase, these molecules elongate and increase the connection direction angles with an increase in the energy level. Li et al. (2002) described the recovery phase when the applied stress is released; the molecules of the examined sample attempt to regain their previous configuration and energy level [22].

Viscoelasticity is the property of constituents that display both viscous and elastic appearances through the deformation stage, exhibiting time-dependent strain. For materials that show only elastic behavior, the extent of distortion is relative to the assigned stress and this magnitude of deformation is sustained if the assigned stress is maintained. After the assigned stress is freed, the present distortion vanishes concurrently as elaborated upon by Ferry (1980) and mentioned by Li et al. (2002) [21,22]. Therefore, elasticity results from the bond extending as a response to the applied stress on an ordered solid structure, whereas viscosity results from the molecular dissemination inside liquid materials due to the effect of the applied shear stress. Dolz et al. (2008) studied the creep and recovery of oil food emulsions with viscoelastic behaviors [23]. They found that all the examined emulsions of their study were characterized by viscous and elastic contours. For materials with viscoelastic activities, the structure experiences distortion within the network restrictions. Then unceasing distortion leads to linkage disassembling. When the assigned stress is freed, the distortion involved both the retained viscous portion and a regained elastic contribution [24].

The viscoelastic behavior of crude oil-Xanthan emulsions is vital for many engineering applications such as in the crude oil industries. The intention of the existing study is to explore the viscous and elastic behaviors for crude oil-gum emulsions. This experimental assessment addresses different crude oil and gum concentrations of two types of Xanthan gums.

## 2. Materials and Methods

### 2.1. Xanthan Materials

Two Xanthan gums were used for this study. The first one is a chemical grade from Sigma-Aldrich Canada Ltd. (Oakville, ON L6H 6J8, Canada), product # G1253 under the product name of Sigma. The second type is an industrial grade of Xanthan gum from CP Kelco (Atlanta, GA 30339, USA) with product # 10040282, and product name of Kelzan. Both gums are white to tan colored powders, and they are recommended to be used for non-food applications such as a thickener and rheology control agents.

### 2.2. Crude Oil

Crude oil from the North Sea was employed in all the experimental measurements with a viscosity of 7.16 mPas at 40 °C, a density of 880.6 kg/m^3^ at 15 °C, and an acid value of 0.0012 kg KOH/kg. The crude oil was supplied by Shell Canada Limited.

### 2.3. Emulsifying Agent

A surfactant material is necessary to prepare stable oil emulsions. It is commonly added into an oil-aqueous phase system as an emulsifying agent to lower the interfacial tension between the oil phase and the aqueous solution medium, and to stabilize the presence of the oil droplets within the aqueous continuous phase [25]. Triton X-100 from Sigma-Aldrich Canada Ltd. was used as a surfactant with a specific gravity of 1.07 and flash point of 113 °C.

### 2.4. Preparation of Crude Oil-Xanthan Emulsions

The Xanthan solutions were prepared first by mixing the Xanthan gum powder slowly in 0.25 L of warm distilled water to the required concentration. The solutions were gently stirred until all the Xanthan gum completely dissolved. Since the Xanthan solution is biodegradable, 1.0 gm formaldehyde was added and the solutions were stored at 4 °C until use to avoid bacterial growth [26,27]. Crude oil-Xanthan gum emulsions were obtained by well mixing of crude oil and Xanthan gum aqueous solution which contains 1 wt. % of Triton X-100 as a surfactant material. A Silent Crusher M homogenizer (Heidolph Instruments GmbH and Co, Schwabach, Germany) was used at a speed of 10^4^ rpm for 5 min to prepare stable crude oil-Xanthan gum emulsions [26].

### 2.5. Rheometer and Measurements 

The creep-recovery experimental study of crude oil-Xanthan emulsions was carried out at a room temperature of 22 °C utilizing the RheoStress RS100 under CS-mode. The software package RS100 was used for operation and measurements of the viscoelastic examination. A cone-plate sensor was employed with a cone diameter of 35 mm, cone angle of 4°-, and an 0.137-mm gap at the cone tip. A water bath was connected to the rheometer to control the applied temperature of the RS100 system [27].

A test sample of 1 cm^3^ was located on the cone-plate sensor, and then the sensor was driven automatically to the precise position. After measurement data was accomplished, the RS100 software package was used to complete the data analysis. A wide range of shear stress of 0.1 to 10 Pa, crude oil concentration range of 0–75% by volume, Xanthan concentration range of 0–10^4^ ppm, and two types of Sigma and Kelzan Xanthan gums were tested. To ensure reliability of the experimental measurements, a selected set of experimental runs were repeated three times for the same emulsion. The measured creep-recovery phases overlay with each other within the permission level of nearly ±2.5%.

## 3. Modelling of Creep and Recovery Behaviors

**Figure 1:**
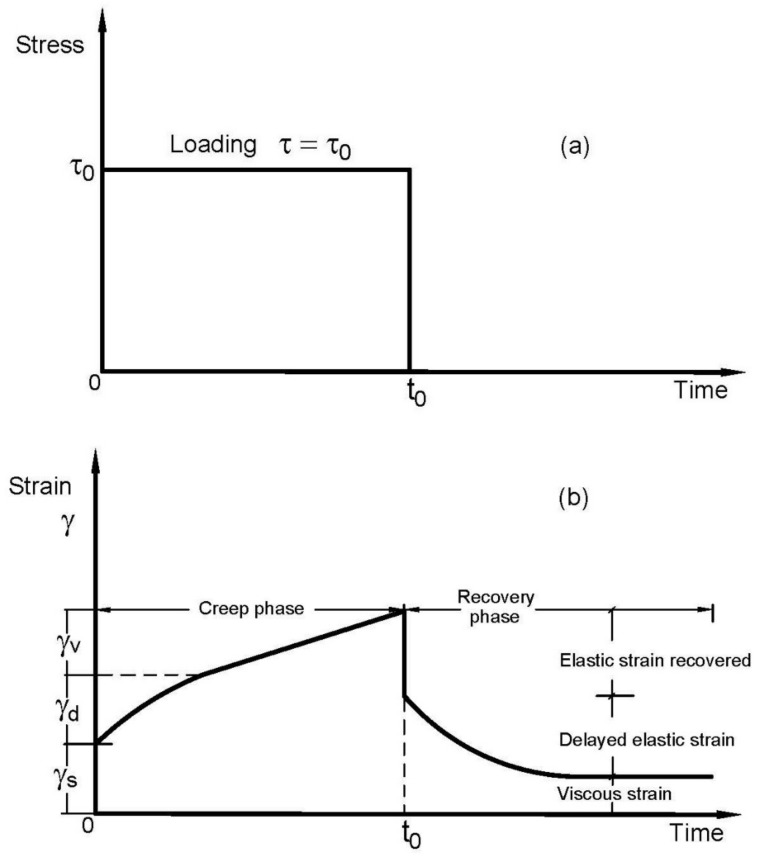
Viscoelastic material curve. (**a**) Loading mode; (**b**) Creep and recovery behaviors.

Creep is a time-dependent deformation, γ, of viscoelastic material due to the effect of constant applied shear stress τ_o_; some of this sheared deformation is recoverable with time after the release of the applied effect. The viscoelastic behavior of a representative material is presented in Figure 1 and characterized by two phases of creep and recovery [23,28]. In Figure 1a, it is shown that a persistent level of stress such as τ_o_ is influenced immediately by the specimen and preserved for a time t_o_, whereas Figure 1b shows the creep-phase and subsequent strain recovery-phase curves for a viscoelastic specimen. In reaction to the applied effect of τ_o_, the time-dependence of creep deformation γ_C_ of viscoelastic behavior can be stated, as in Equation (1) in which γ_s_ is the immediate strain, γ_d_ (t) is the delayed strain, and γ_v_(t) corresponds to the viscous contribution. For the linear viscoelastic response, the γ_s_ denotes the elastic contribution of the specimen, which is reversible, disappears on the elimination of the applied stress and can be displayed by γ_elastic_. The second constituent of the creep strain is γ_d_ (t), which refers to the delay elastic strain with decreasing rate over time and requires time for the complete recovery. This contribution can be referred to the chain uncoiling [21]. The third constituent γ_v_ (t) represents the viscous flow contribution of the specimen, which is an irreversible part of the strain and increases linearly with time for the linear viscoelastic materials.

γ_C_ (t) = γ_s_ + γ_d_ (t) + γ_v_ (t) (1)

Under the applied constant shear stress τ_o_, materials with viscoelastic response exhibit a time-dependent deformation profile of γ_C_ (t) as:

γ_C_ (t) = τ_o_ J_C_ (t) (2)

J_C_ (t) represents the material compliance; it exhibits the material deformation at a definite assigned stress. By introducing Equation (2) into Equation (1), the creep compliance J_C_ (t) can be stated as:

J_C_ (t) = γ_C_ (t)/τ_o_ = J_instantaneous elastic_ + J_delay elastic_ (t) + J_viscous_ (t) (3)

Once the stress τ_o_ is released at time t_o_, the recovery phase shows an instantaneous contraction of the immediate elastic strain recovered at t_o_, followed by a period of slow recovery of the delayed elastic strain over elapsed time, whereas the viscous strain rests unrecovered and can be stored as perpetual deformation in the specimen. Therefore, the time-dependence of strain recovery γ_R_ (t) can be presented as:

γ_R_ (t) = γ_elastic_ + γ_slow recovery_ (t) (4)

Correspondingly, the J_R_ (t) can be identified as:

J_R_ (t) = γ_R_ (t)/τ_o_ = J_elastic_ + J_slow recovery_ (t) (5)

**Figure 2:**
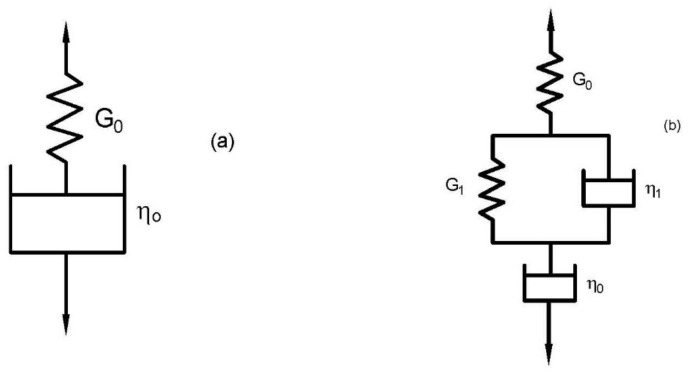
Maxwell and Burger models. (**a**) Maxwell model; (**b**) Burger model.

The creep phase of the polymer solutions can be imitated through the Maxwell- or Burger-model reliant on the presence of J_delay elastic_ (t) contribution within the reported behavior. The Maxwell model comprises of a coil with modulus G_o_ and a dashpot with viscosity η_o_ arranged in the sequence manner as in Figure 2a. The main contributions of the Maxwell equation can be presented as [24]:

J_C_ (t) = (1/G_o_) + (1/η_o_) t (6)

On the other hand, the Burger model involves two coils with moduli G_o_ and G_1_, and two dashpots with viscosities η_o_ and η_1_. This model is a series combination of the Maxwell and Kelvin–Voigt models arranged as in Figure 2b. The Burger model can be formed by the following mathematical model [24]:

J_C_ (t) = (1/G_o_) + (1/η_o_) t + (1/G_1_) (1−exp (−t/λ_1_)) (7)

## 4. Results and Discussion

### 4.1. Flow Behavior of Oil-Xanthan Emulsions

The flow behavior of crude oil-Xanthan emulsions was explored in relations of viscosity and shear stress versus shear rate [27]. It has been reported that the flow behaviors of these emulsions rely upon the shear rate, kind of the added gum, and concentration of the added crude oil and gum. The Xanthan solutions of both tested types showed non-Newtonian with shear thinning profile in which the apparent viscosity diminished with shear rate. Similar flow behaviors were reported by Yeon et al. (2014) and Khan et al. (2018) [29,30]. The flow manner of crude oil-Xanthan mixture is characterized by non-Newtonian with shear thinning response [16,17,18]. The measured viscosity of the examined emulsions revealed that the flow behavior relies primarily on the shear rate and Xanthan gum addition. Jha et al. (2018 and 2021) studied the effect of polymers on the rheological properties of oil-water emulsions, and the impact of Xanthan gum on the development emulsion drilling fluids with strong shear thinning behavior were concluded [31,32]. The impact of gum addition is more manifest at the lower range of shear rate. Nevertheless, at greater shear rate the hydrodynamic result is more dominant than the gum concentration. In addition, it has been found that the Casson model, Equation (8), can be used to fit the reported measurements of the investigated emulsions over the examined concentration range [27].

(8) $$\tau =(\tau_{{\mathrm{app}}^{0.5}}+(\dot{\gamma }\eta_{c}{)}^{0.5}{)}^{2}$$

where τ is the shear stress in Pa, τ_app_ is the apparent yield stress in Pa, $\dot{\gamma }$ is the shear rate in s^−1^, and η_c_ is the apparent viscosity in Pa.s. The apparent viscosities of the crude oil-Xanthan emulsions are higher than their corresponding solutions. For the lighter concentration of crude oil (≤25%) and gum addition (≤1000 ppm), both kinds of gum emulsions showed comparable viscosity profiles. However, for the higher concentrations of crude oil and gum, the Kelzan emulsions exhibited slightly higher viscosity profiles than the Sigma emulsions [27].

### 4.2. Linear Viscoelastic Range of the Examined Emulsions

When a tested sample with viscoelastic behavior is exposed to constant shear stress of τ_o_, the emulsion reveals time-dependent strain behavior with compliance responses as displayed by Equation (2). The higher the values of the emulsion compliance display, the easier the liquid can be strained. The behavior of the emulsion compliance depends upon the level of the applied shear stress. At the lower values of the applied shear stress, the viscoelastic material usually shows a rectilinear profile. However, for the higher shear stresses, the viscoelastic profile exhibits a non-linear behavior in which the test results rely on the settings and considerations of the instrument [33]. Moreover, within the linear limits, the applied shear stress will not be destructive, and the distorted consequences can be recalled if the assigned shear is removed, which reveals that the sample structure was elastically stressed while the structure remained undamaged. Consequently, inside the linear limits, the compliance behavior will not rely on the assigned shear [33].

It is necessary to find the shear stress range which results in linear viscoelastic behavior for the crude oil-gum emulsions. The following test was carried out for different assigned shear stresses, gradually ascending in order to find the linear limitations. Firstly, the emulsion sample was placed in the right position inside the RS100 unit; then, a constant shear stress of a low value of 0.1 Pa is applied for a period of 250 s to check the creep phase. The assigned shear is then removed to follow the recovery profile for another period of 250 s. Similar examining tests were repeated for new emulsion samples of the same characteristics, but at higher applied shear stresses. Variety of shear stresses over the range of 0.1–10 Pa were applied. If the compliance results of various assigned shear stresses overlapped each other, this provided the linear viscoelastic range. On the opposite side, the non-linear domain caused the compliance profiles to diverge considerably from the linear profiles [33,34]. 

**Figure 3:**
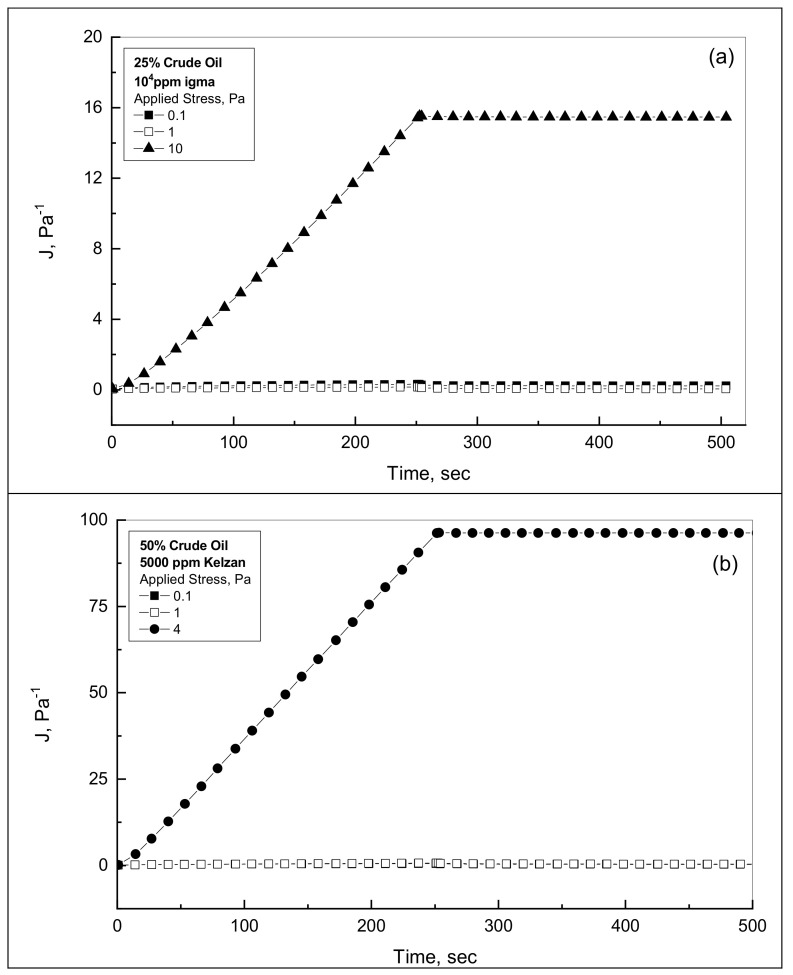
Compliance behavior for two different emulsions. (**a**) 25% oil-10^4^ ppm Sigma emulsion; (**b**) 50% oil-5000 ppm Kelzan emulsion.

Many experimental runs have been completed for different emulsions with different applied shear stresses to determine the stress range that establishes the linear viscoelastic range. Figure 3a,b shows typical examples of these tests for 25% and 50% crude oil in the presence of Sigma and Kelzan gum. These investigations of the creep-recovery behaviors under different assigned shear stresses up to 10 Pa in Figure 3a,b show that the assigned stresses up to 1.0 Pa cause linear profiles for the whole tested crude oil-Xanthan emulsions. Dolz et al. (2008) studied the linear viscoelastic region of some oil-Xanthan emulsions by using the stress sweep between 0.1 and 100 Pa, and they recorded the creep tests at a constant stress amplitude of 3 Pa for emulsions [23]. However, Pal (1996) carried out creep-recovery tests for polymer thickened oil-water emulsions at a constant stress amplitude of ≤0.5 Pa [35]. Thus, unless it is cited otherwise, all the further experimental runs of viscoelastic behavior are tested at an assigned stress of 0.1 Pa to confirm that the structure of the emulsions can be elastically strained, whereas the sample structure remains unbroken [34].

### 4.3. Effect of Xanthan Concentration

**Figure 4:**
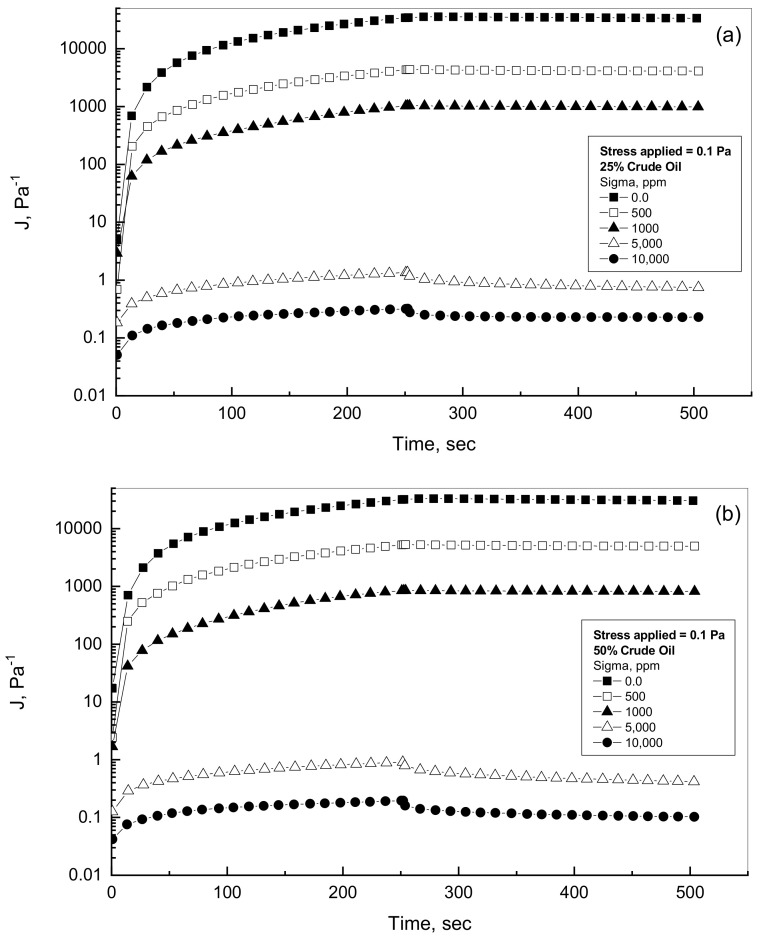
Creep-recovery behavior for 25% and 50% crude oil-Sigma emulsions. (**a**) 25% oil-Sigma emulsions; (**b**) 50% oil-Sigma emulsions.

To address the effect of the gum addition on the viscoelastic performance in terms of creep and recovery of the prepared emulsions, a wide range of Sigma addition over the range 0–10^4^ ppm was tested for different oil emulsions. Figure 4a,b displays typical examples for the examined emulsions in terms of compliance against time. As can be observed from Figure 4a,b, the Sigma addition commands the performance and the magnitude of the crude oil-Xanthan results. The higher the addition of the tested gum, the lower the compliance results of the emulsion. For each emulsion, if the assigned constant shear stress is applied, the compliance measurement of Sigma emulsions increases gradually with time within the creep phase. This behavior reached its peak at the end of the creep phase, i.e., at the end of 250 s. Within the creep phase, the time rate of compliance change decreased over the elapsed time. This behavior can be explained through the tested emulsion, which experienced an increase in creep resistance. Therefore, the deformation of the tested emulsion became more difficult as the material was stressed [22,23,33].

Once the creep mode was completed at the end of 250 s, the allocated shear stress was removed, and the recovery phase was observed versus elapsed period for the subsequent 250 s, as can be assessed in Figure 4. Table 1 shows the compliance measurements at the end of creep phase J_250_, and at the end of recovery phase, J_500_, for the 25% and 50% crude oil-Sigma gum emulsions. As listed in Table 1, the compliance values decline significantly with the addition of gum and crude oil concentrations. These remarks are in a full agreement with the results reported by Pal (1996) in which the polymer and crude oil concentrations dictate the viscoelastic behavior in terms of creep and recovery [35]. The addition of crude oil into the polymer emulsions leads to lower creep-recovery behavior and consequently higher elastic response [35].

**Figure 5:**
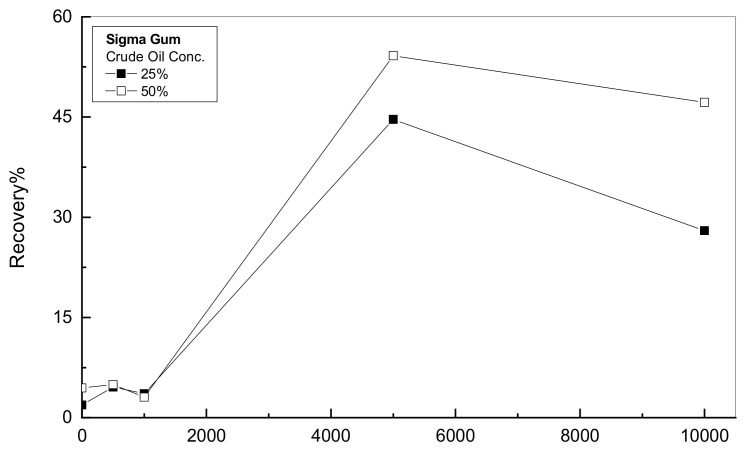
Recovery behavior for crude oil-Sigma emulsions.

As discussed earlier, it is necessary to address the recovery achieved by the emulsion at the end of each test. The compliance measurements can be utilized to anticipate the magnitude of the recovery that can be sustained by using Equation (9) taking into consideration the differences between the reported values of deformation at the creep end and the deformation at the recovery end w.r.t. the deformation at the end of the creep phase [23]. Figure 5 illustrates the recovery percentage versus the Sigma concentration for two crude oil concentrations of 25% and 50%. In general, the recovery% enhances gradually with both the crude oil and gum concentrations up to 5000 ppm. The recovery % of the tested emulsions reveals the elastic contribution of the oil-gum emulsion. Figure 5 shows that the oil-gum emulsions with gum addition of up to 1000 ppm display only viscous behavior since the recovery % is very near zero. The recovery % of the higher concentrations of both gum and oil exhibits greater recovery, which indicates viscoelastic behavior. Therefore, the presence of polymer material and crude oil boosts the elastic contribution of the tested emulsions as reported by Pal (1996) [35].

Recovery% = (Creep end value−Recovery end value) × 100/Creep end value(9)

### 4.4. Effect of Crude Oil Concentration

**Figure 6:**
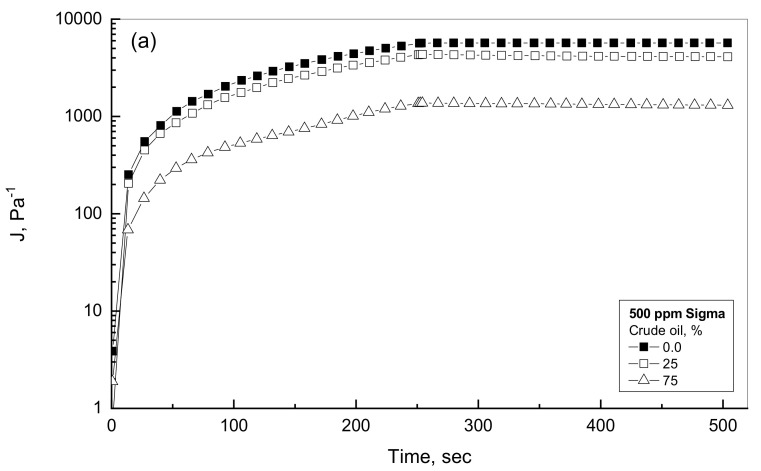

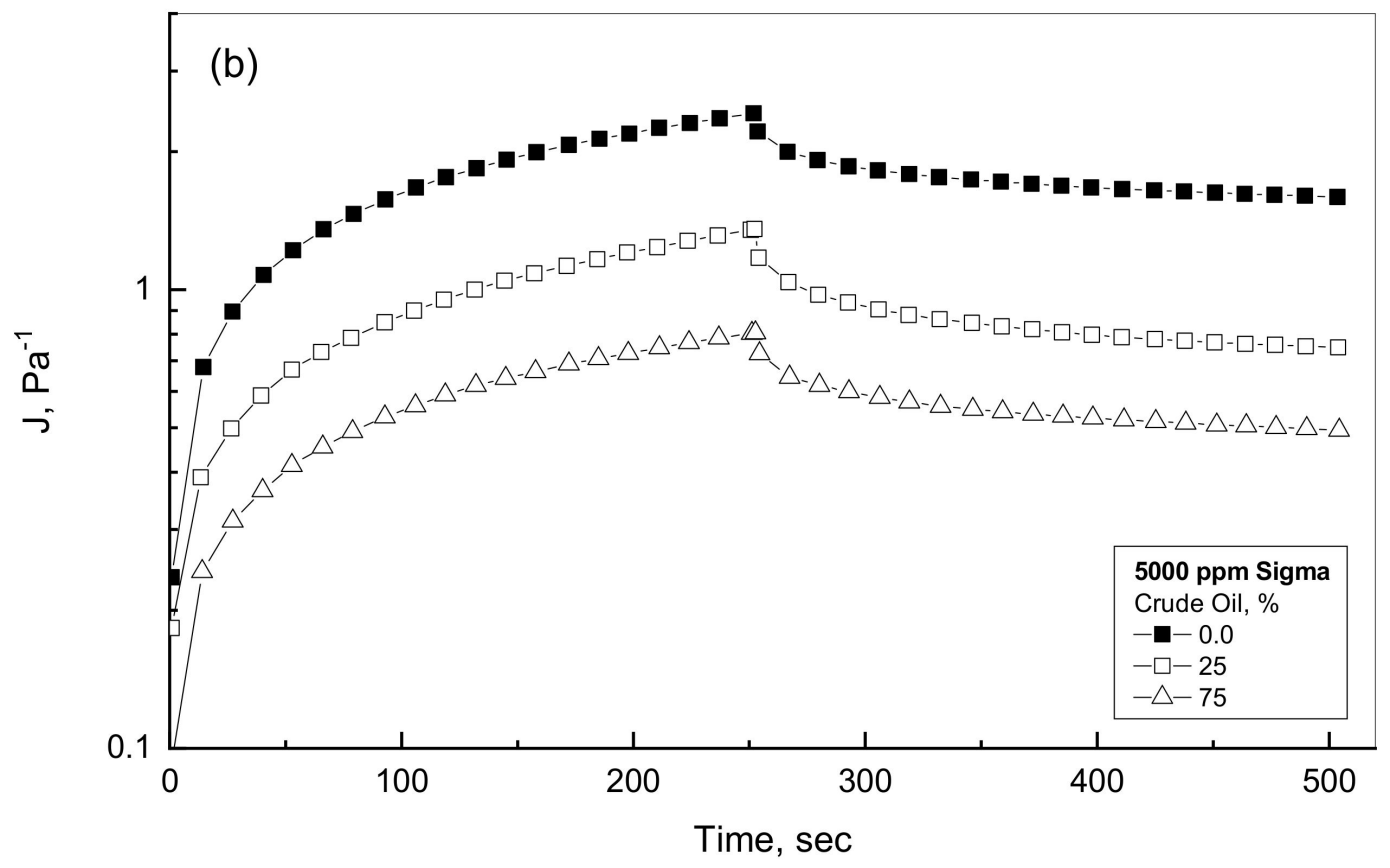
Effect of oil addition for 500 and 5000 ppm of Sigma emulsions. (**a**) Low Sigma concentration; (**b**) High Sigma concentration.

The aim of this investigation is to search for the role of oil presence within the emulsion in viscous and viscoelastic diverse situations. Figure 6a illustrates the creep and recovery behaviors for the 0, 25%, and 75% of oil addition into 500 ppm solution as a typical example for low Sigma gum concentration. Figure 6a shows viscous performance for the 500-ppm solution, i.e., without crude oil. For this case, the compliance value reaches 5650 The availability of crude oil inside the Sigma gum solution progressively decreases the measured compliance values."Pa^−1^ at the end of creep phase of 250 s. The availability of crude oil inside the Sigma gum solution declines progressively with the measured compliance values. For example, the compliance value at the end of the creep phase reaches 4305 and 1362 Pa^−1^ for 25% and 75% oil addition. Both of these examined emulsions exhibit recovery % of less than 5% at the end of the recovery phase. Therefore, the three tested cases of the 500-ppm gum solution and its emulsions of 25 and 75% crude oil addition display only viscous behavior since the reported recovery % is very limited. Pal (1996) concluded only viscous behavior for oil-polymer emulsions with an oil concentration of less than 65% by volume [35].

It is necessary to examine the similar profiles for the higher Sigma gum concentration. Figure 6b, for the 5000 ppm Sigma solution, illustrates the viscoelastic behavior of the gum solution and two more crude oil-Sigma emulsions. These tested cases of high gum concentration demonstrate much lower values of the measured compliance associated with higher elastic recovery portion in comparison with the viscous environment case displayed in Figure 6a. The existence of oil regularly depresses the compliance results and slightly surges the recovery contribution. For example, the measured compliance values at the end of creep phase are 2.424, 1.351 and 0.803 Pa^−1^ for 0, 25% and 75% oil content, as can be observed in Figure 6b. The recovery % according to Equation (9) for 0, 25% and 75% emulsions are 34.3%, 44.6% and 38.5%, respectively. Therefore, the addition of crude oil into higher gum concentration of 5000 ppm enhances the elastic activities of the crude oil-Sigma gum emulsions. The existence of crude oil droplets phase within the aqueous phase of the Xanthan gum enhances the apparent viscosity of the emulsions, which can be contributed to the interactions and collisions between the oil droplets and the polymer network [27]. The viscoelastic behavior of the examined emulsions is in agreement with the characteristics of the flow profile discussed earlier, owing to the presence of oil phase. Sosa–Herrera et al. (2008) reported that the elastic response of emulsion is higher than that of the polymer solution, which is due to the presence of suspended oil droplets within the polymer solution [36,37].

The surface charges of the polysaccharide molecules allow for the interconnection between the oil surface and the molecules of the Xanthan gum [38]. Thus, an interfacial film establishes through either hydrogen bonds, van der Waals or electrostatic interactions, providing oil-gum stabilized emulsion [39,40]. Numerous studies have specified that the hydrogen bonding and electrostatic interactions display a significant effect on the oil-gum network structure [41]. Jiang et al. (2020) reported that the hydrogen bonding and electrostatic interactions of the oil-gum emulsion form pseudoplastic fluids with oil elastic behavior [42]. Therefore, the viscoelastic response of the crude oil-gum emulsions can be attributed to the viscoelasticity behavior of the high concentration gum solution continuous phase, which is a viscoelastic in nature, the droplets dispersed phase of the high concentration of the crude oil which exhibits some shape elasticity, and the intermolecular interactions between the oil droplets dispersed phase and the polymer networks structure.

### 4.5. Comparison of Sigma and Kelzan Creep-Recovery Behavior

**Figure 7:**
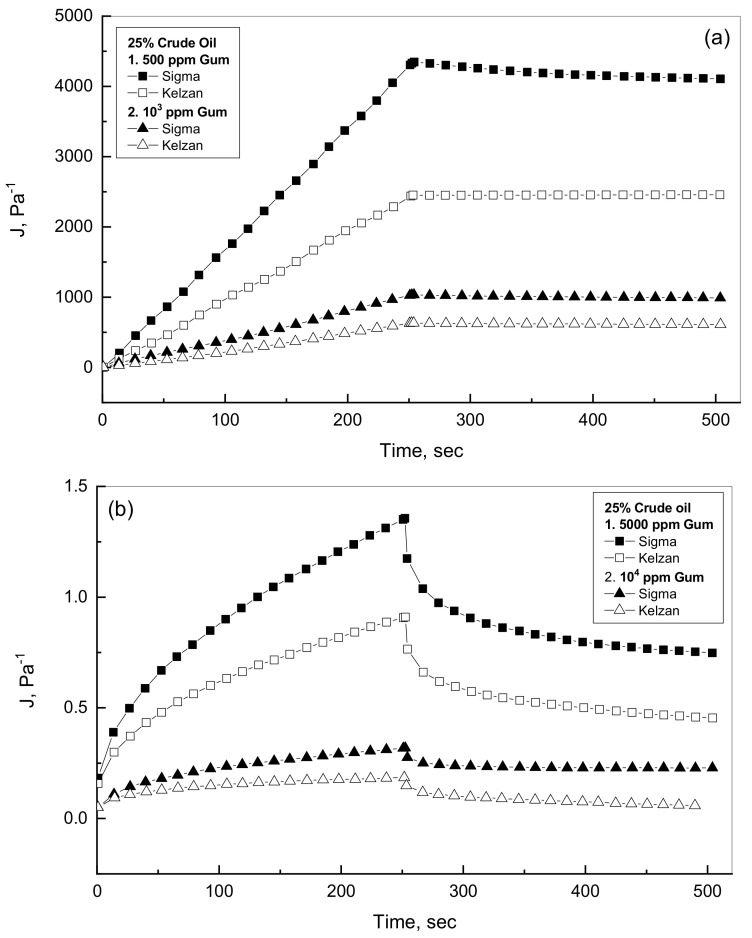
Creep-recovery behavior of Sigma and Kelzan for 25% oil emulsions. (**a**) Low Xanthan concentration; (**b**) High Xanthan concentration.

**Figure 8:**
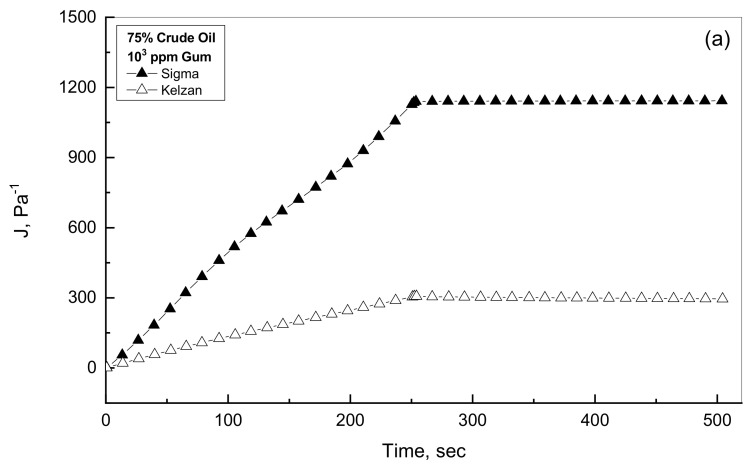

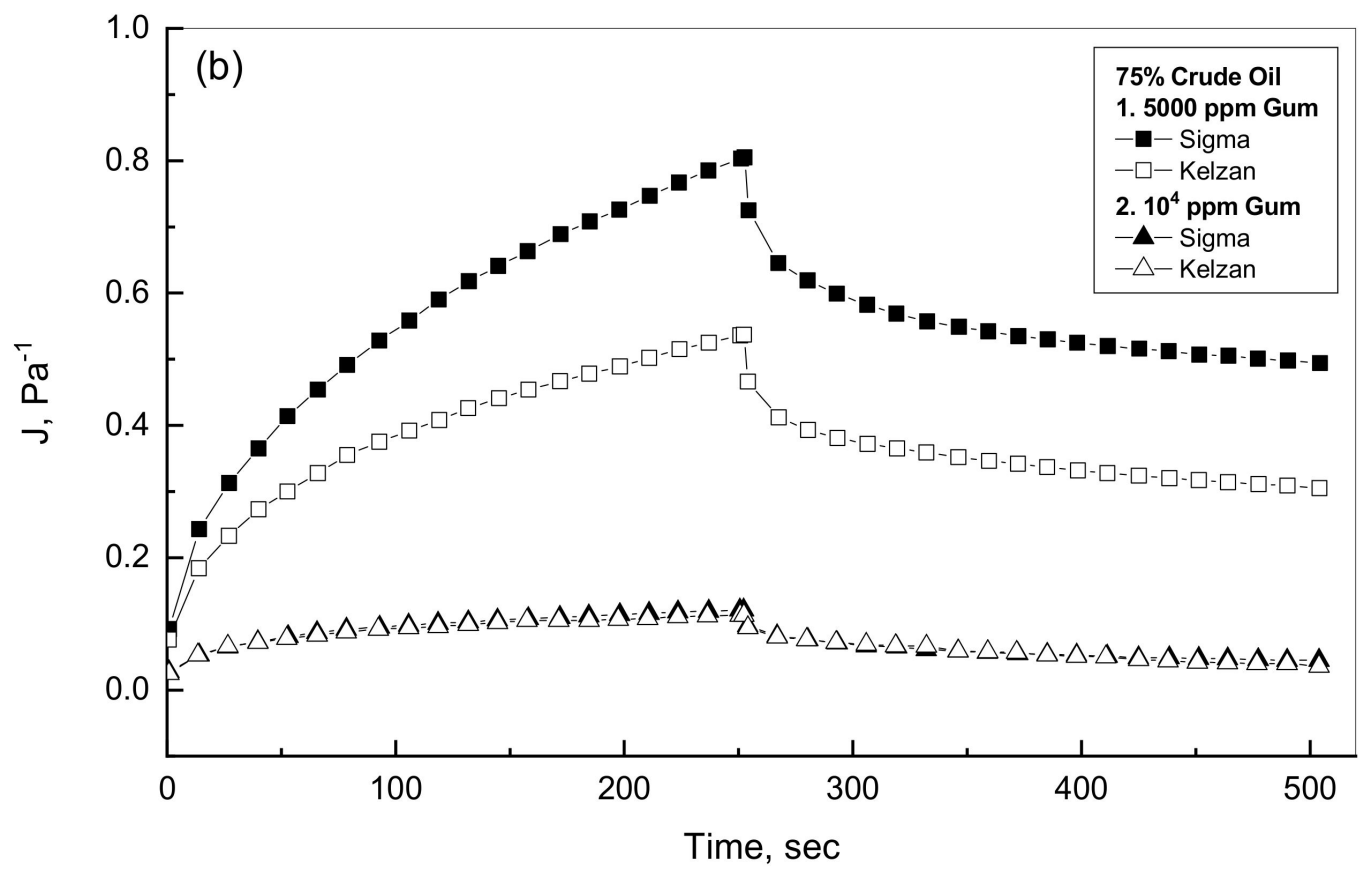
Creep-recovery behavior of Sigma and Kelzan for 75% oil emulsions. (**a**) Low gum concentration; (**b**) High gum concentration.

It is valuable to investigate the viscoelastic characteristics of oil-gum mixtures in the presence of two different gums such as Kelzan and Sigma. This study observed variety of selected additions of oil and gum. Figure 7a,b shows the compliance profiles for low- and high-additions of Sigma and Kelzan in the presence of little concentration of 25% crude. Figure 7a displays almost linear behavior for both of creep and recovery phases for the low gum concentration of up to 1000 ppm with Sigma emulsions considerably higher profiles than the Kelzan emulsions. For the low gum concentration of up to 1000 ppm, Figure 7a reveals very low recovery % by using the compliance results at the expiration of both creep and recovery phases. This kind of creep-recovery profile for the low gum concentrations can be considered viscous behavior as discussed before [36,43]. Figure 7b shows the creep-recovery behavior for the higher gum concentration range in the existence of 25% crude oil. As can be described from Figure 7b, much less compliance values with non-linear profiles for both creep and recovery phases reveal viscoelastic characteristics for both tested Xanthan gums. This observation can be referred to the viscoelasticity nature of the high gum concentrations, the crude oil dispersed phase and the intermolecular interactions of the oil droplets medium inside the Xanthan gum network formed by the high concentration. This means that the elastic characteristic improves considerably with further addition of the Xanthan gum, leading to the formation of gel-like behavior [36,43]. Similar behaviors are observed in Figure 8a,b for the low- and high-Xanthan gum additions in the presence of 75% crude oil concentration. 

**Figure 9:**
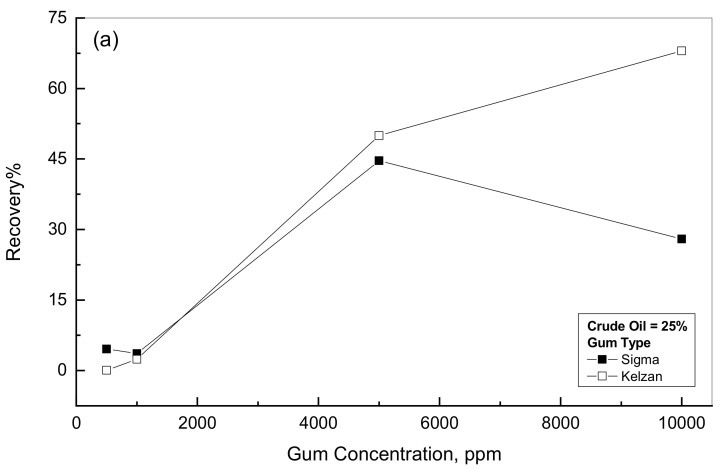

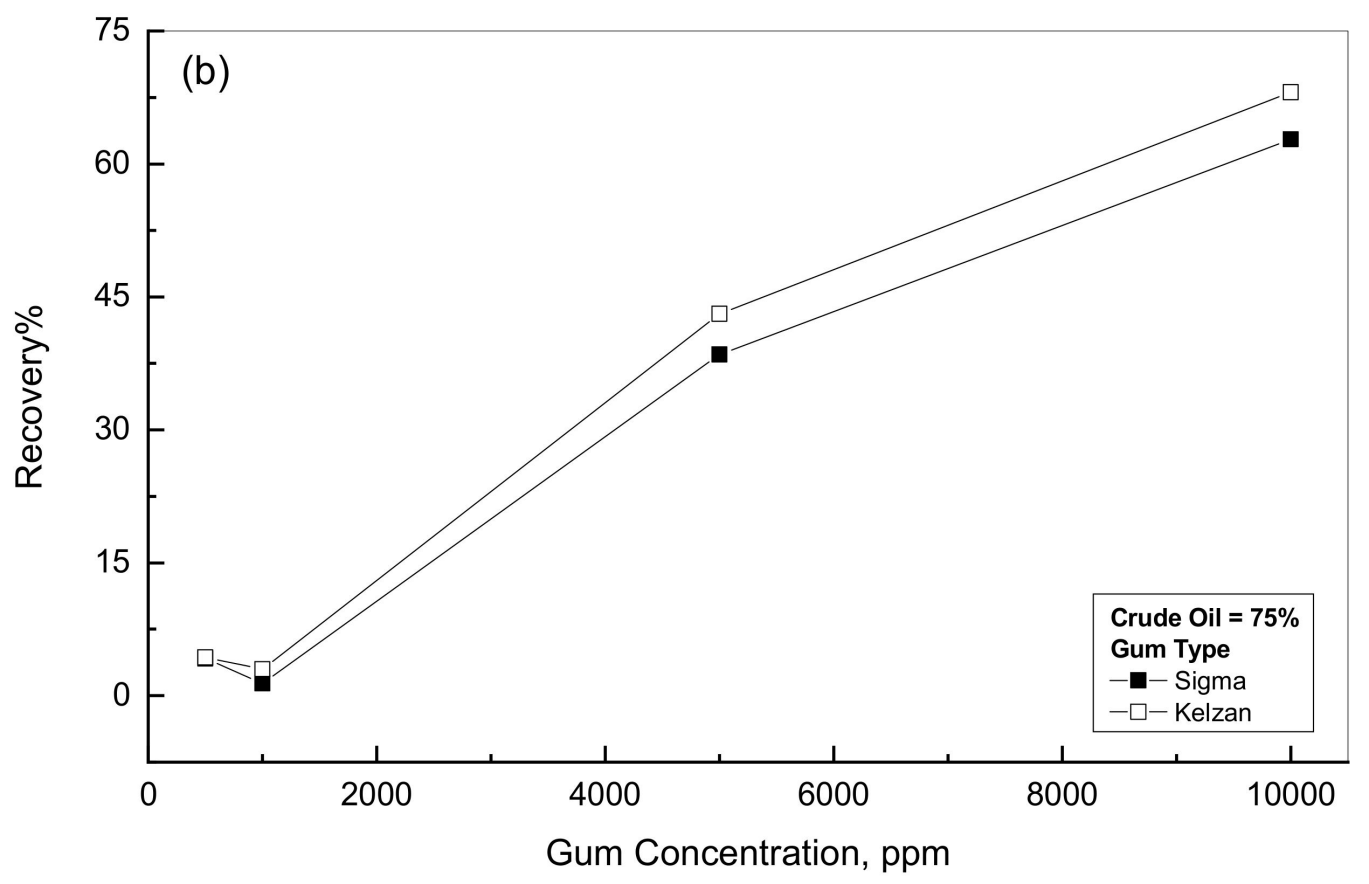
Recovery behavior for 25% and 75% crude oil-gum emulsions. (**a**) Low crude oil concentration; (**b**) High crude oil concentration.

The recovery % analysis was completed for the examined oil-gum emulsions with various additions of Sigma and Kelzan. Figure 9 exhibits the analysis of the recovery % versus the gum concentration for the two tested Sigma and Kelzan. In general, for gum concentration higher than 1000 ppm, the recovery % increases considerably with gum addition and oil concentration. One exception for this behavior is the 25% crude oil-Sigma emulsion of 10^4^ ppm concentration; it shows a slight drop in recovery % from its peak at 5000 ppm. Figure 9a,b show that the Kelzan emulsions display higher recovery % than the Sigma emulsions. The recovery % of the examined emulsions depicts the elastic portion within the crude oil-gum emulsions. It is obvious from Figure 9 that the lower concentrations than 1000 ppm of both types of gums emulsions can be considered only viscous behavior since their recovery % is very close to zero for both materials. This conclusion is in agreement with the results reported by the Akiyama et al. (2005) [43]. The presence of high polymer concentration within oil emulsion forms some kind of gel status through the associated networks of polymer molecules during the intermolecular interactions [44]. However, for the diluted polymer solutions, the connected network will not be available. Therefore, the higher concentrations of Xanthan gums emulsions show much higher recovery %, which indicates viscoelastic behavior [45].

From the earlier discussion and illustrations of the creep behavior, it is obvious that the oil-gum mixtures of light Xanthan content (i.e., less than 5000 ppm) perform in almost full viscous behavior and will be modelled by Equation (6). Whereas the higher gum concentrations of the oil emulsions exhibit instantaneous-, delayed elastic-, and viscous flow-deformations of viscoelastic behavior. Consequently, these higher gum concentrations of oil emulsions can be demonstrated sufficiently with Equation (7). Additionally, these remarks can be acquired from the examination of the Maxwell- and Burger-models. The outcomes of these models are listed in Table 2 and Table 3 with the regression coefficient R^2^. Table 2 displays the Maxwell factors of G_o_ and η_o_ for lower concentrations of Xanthan gums with variety of crude oil addition, while the next table reports the Burger factors of G_o_, G_1_, η_o_, and η_1_ for the higher Xanthan additions in the presence of diverse oil content. Table 2 shows the dashpot contribution of the viscosity η_o_ values and almost a nonexistence of the coil involvement of the Maxwell model except for the 75% oil-10^3^ ppm emulsion, which displays a very minor contribution of the G_o_. Therefore, the selection and estimates of the Maxwell model support the acquired remarks of the complete viscous behavior of the lower gum emulsions of less than 5000 ppm Xanthan concentrations.

Table 3 displays the viscoelastic modelling parameters which enhance considerably with the concentration, i.e., the viscoelastic characteristics growth significantly with emulsion concentrations of crude oil and gum additions. In general, owing to the presence of the elastic contributions of G_o_ and G_1_ listed in Table 3; therefore, the expectation of the Burger model agrees very well with the prior remarks of the viscoelastic profiles of the higher gum and oil emulsions. For all the tested emulsions, the four parameters of the Burger model presented similar trend with oil and gum concentrations. The elastic moduli G_o_ and G_1_ of the Burger model increase significantly with concentrations of both phases in which G_o_ is higher than G_1_. Therefore, the values of the instantaneous elastic modulus (G_o_) are higher than the predicted values of the retarded elastic region (G_1_) for all the tested emulsions.

The recovery segment was observed when the applied stress τ_o_ is removed at time 250 s and then the recovery phase was recorded over the period 250–500 s. The recovery phase of the viscoelastic crude oil-gum emulsions displays a sudden shrinkage of the instant elastic strain recovered at 250 s and corresponds to the Maxwell model coil modulus (G_o_). The second part of recovery is slower, and gradual recapture of the deferred elastic strain with time. This gradual exponential recovery corresponds to the Kelvin–Voigt modulus (G_1_). The third part of recovery is the viscous contribution which lasts unrecaptured and dissipated within the specimen [21]. Consequently, the time-dependence of J_R_ (t) can be displayed by Equation (10) as:

J_R_ (t) = (1/G_o_) + (1/G_1_) exp (−t/λ_1_) (10)

The modelling investigation utilizing Equation (10) was implemented to find the appropriate parameters to describe the recovery segment of the various crude oil-gum emulsions. The outcomes of this examination are listed in Table 4 and are associated with the regression factor of R^2^. Table 4 shows that the modelling parameters of Equation (10) grow gradually with further addition of both crude oil and gum materials, enhancing the recovery % behaviors of the crude oil-gum emulsions as indicated earlier in Figure 9. For all the examined emulsions, Table 4 displays that the instantaneous elastic recovery of G_o_ is much higher than the gradual recovery contribution. 

## 5. Conclusions

This experimental study investigates the viscous and elastic behaviors of crude oil-Xanthan gum emulsions. The gum and oil concentrations dictate the characteristics of the creep-recovery curve and the magnitude of the measured compliance. The presence of the crude oil within the gum solution declines the measured compliance for both of creep and recovery behaviors. For the gum concentrations of higher than 10^3^ ppm, the crude oil-gum emulsions display non-linear of viscoelastic behaviors due mainly to the gum viscoelastic responses and the intermolecular interactions between the crude oil droplets and the Xanthan gum networks formed within the emulsions. Furthermore, the addition of crude oil enhances the elastic contribution of the tested emulsions. The reported creep-recovery profiles of the crude oil-Sigma emulsions are slightly higher than the emulsions of Kelzan. However, for the lower gum addition of less than 10^3^ ppm, both types of gums emulsions display linear creep-recovery profiles of viscous behaviors. In general, crude oil-Kelzan emulsions demonstrate recovery % slightly higher than the crude oil-Sigma emulsions. Maxwell and Burger models were utilized to model both of the viscous and viscoelastic profiles of the tested emulsions. Although further studies are necessary to assess the viscoelastic behavior of the crude oil and gum emulsions, the remarks and measurements of the current study will be useful knowledge to utilize Xanthan gums in the enhanced oil recovery. 

## Figures and Tables

**Table 1:** Compliance measurements at the end of creep and recovery modes.

	25% Oil		50% Oil	
Sigma, ppm	J_250_, Pa^−1^	J_500_, Pa^−1^	J_250_, Pa^−1^	J_500_, Pa^−1^
0	34,221	33,571	31,848	30,433
500	4304	4107	5211	4953
1000	1026	989	848	818
5000	1.351	0.748	0.91	0.417
10,000	0.318	0.229	0.195	0.103

**Table 2:** Creep modelling using Maxwell equation for lower gum emulsions.

*A-Sigma Xanthan*									
	25% Oil			50% Oil			75% Oil		
Gum Conc.	G_o_	η_o_	R^2^	G_o_	η_o_	R^2^	G_o_	η_o_	R^2^
	Pa	Pas		Pa	Pas		Pa	Pas	
500 ppm	0.0	0.058	0.99	0.0	0.048	0.99	0.0	0.188	0.99
10^3^ ppm	0.0	0.245	0.99	0.0	0.288	0.99	0.045	0.227	0.99
***B-Kelzan Xanthan***									
	25% Oil					75% Oil			
Gum Conc.	G_o_		η_o_	R^2^		G_o_	η_o_		R^2^
	Pa		Pas			Pa	Pas		
500 ppm	0.0		0.102	0.99		0.0	0.059		0.99
10^3^ ppm	0.0		0.393	0.99		0.089	0.845		0.99

**Table 3:** Creep modelling using Burger equation for higher gum emulsions.

		G_o_	η_o_	G_1_	η_1_	R^2^
		Pa	Pas	Pa	Pas	
Gum Conc.			***A. 25% Crude Oil***			
5000	Sigma	5.3	333.3	2.4	83.2	0.999
10^4^	Sigma	19.8	1666.7	7.8	240.0	0.999
Gum Conc.			***B. 50% Crude Oil***			
5000	Sigma	7.8	526.3	3.3	103.6	0.998
10^4^	Sigma	23.8	3333.3	12.5	426.2	0.999
Gum Conc.			***C. 75% Crude Oil***			
5000	Sigma	10.2	625	3.2	117.7	0.998
10^4^	Sigma	36.2	5000	17.9	537.2	0.999
Gum Conc.			***D. 25% Crude Oil***			
5000	Kelzan	6.3	555.6	3.4	117.5	0.999
10^4^	Kelzan	19.7	5000	12.1	333	0.995
Gum Conc.			***E. 75% Crude Oil***			
5000	Kelzan	5.2	666.7	114.9	0.012	0.910
10^4^	Kelzan	39.7	10,000	17.9	470.0	0.994

**Table 4:** Results of recovery analysis using Equation (10) for higher gum emulsions.

	*25% Crude Oil*				*75% Crude Oil*			
	G_o_	G_1_	η_1_	R^2^	G_o_	G_1_	η_1_	R^2^
	Pa	Pa	Pas		Pa	Pa	Pas	
5000 Sigma	1.29	0.003	0.103	0.95	1.96	0.008	0.310	0.95
10^4^ Sigma	4.35	0.005	0.221	0.92	20.83	0.079	3.720	0.94
5000 Kelzan	2.08	0.004	0.141	0.93	3.13	0.016	0.677	0.93
10^4^ Kelzan	14.79	0.044	2.017	0.92	28.25	1.118	106.45	0.94

## Data Availability

Not applicable.
